# Heterogeneity of Multiple Sclerosis Lesions in Multislice Myelin Water Imaging

**DOI:** 10.1371/journal.pone.0151496

**Published:** 2016-03-18

**Authors:** Tobias Djamsched Faizy, Christian Thaler, Dushyant Kumar, Jan Sedlacik, Gabriel Broocks, Malte Grosser, Jan-Patrick Stellmann, Christoph Heesen, Jens Fiehler, Susanne Siemonsen

**Affiliations:** 1 Department of Diagnostic and Interventional Neuroradiology, University Medical Center Hamburg-Eppendorf, Hamburg, Germany; 2 Department of Neurology, University Medical Center Hamburg-Eppendorf, Hamburg, Germany; 3 Institute of Neuroimmunology and MS (INIMS), University Medical Center Hamburg-Eppendorf, Hamburg, Germany; Brighton and Sussex Medical School, UNITED KINGDOM

## Abstract

**Purpose:**

To assess neuroprotection and remyelination in Multiple Sclerosis (MS), we applied a more robust myelin water imaging (MWI) processing technique, including spatial priors into image reconstruction, which allows for lower SNR, less averages and shorter acquisition times. We sought to evaluate this technique in MS-patients and healthy controls (HC).

**Materials and Methods:**

Seventeen MS-patients and 14 age-matched HCs received a 3T Magnetic Resonance Imaging (MRI) examination including MWI (8 slices, 12 minutes acquisition time), T2w and T1mprage pre and post gadolinium (GD) administration. Black holes (BH), contrast enhancing lesions (CEL) and T2 lesions were marked and registered to MWI. Additionally, regions of interest (ROI) were defined in the frontal, parietal and occipital normal appearing white matter (NAWM)/white matter (WM), the corticospinal tract (CST), the splenium (SCC) and genu (GCC) of the corpus callosum in patients and HCs. Mean values of myelin water fraction (MWF) were determined for each ROI.

**Results:**

Significant differences (p≤0.05) of the MWF were found in all three different MS-lesion types (BH, CEL, T2 lesions), compared to the WM of HCs. The mean MWF values among the different lesion types were significantly differing from each other. Comparing MS-patients vs. HCs, we found a significant (p≤0.05) difference of the MWF in all measured ROIs except of GCC and SCC. The mean reduction of MWF in the NAWM of MS-patients compared to HCs was 37%. No age, sex, disability score and disease duration dependency was found for the NAWM MWF.

**Conclusion:**

MWF measures were in line with previous studies and lesions were clearly visible in MWI. MWI allows for quantitative assessment of NAWM and lesions in MS, which could be used as an additional sensitive imaging endpoint for larger MS studies. Measurements of the MWF also differ between patients and healthy controls.

## Introduction

Multiple sclerosis (MS) is the most common chronic central nervous system (CNS) disorder of young adults, leading to demyelination and axonal damage [1,2]. Therefore, specific imaging biomarkers are urgently required for early detection of neuroprotective therapy effects and remyelination processes in MS [3]. Imaging markers such as discrete inflammatory lesions in T2-weighted magnetic resonance imaging (MRI) and contrast enhancing lesions in T1-weighted MRI have become biomarkers for the measurement of treatment effects targeted at multifocal inflammatory demyelination [4,5]. Other experimental MRI techniques such as magnetization transfer ratio (MTR) imaging [6,7] or the fractional anisotropy (FA) derived from diffusion tensor imaging (DTI) are non-specific to myelin and are influenced nonlinearly by a multitude of processes affecting the tissue microstructure and biochemistry [8,9].

In contrast, myelin water imaging (MWI) is an imaging technique primarily based on multi echo spin echo (MESE) T2 relaxometry, which may be less sensitive to concomitant pathological processes such as inflammation [10]. Concomitantly, the term myelin water fraction (MWF) has been established to be a potential marker for myelin integrity [11].

Former studies reported reduced MWF in the normal appearing white matter (NAWM) of MS-patients in comparison to healthy controls (HC) in the range of 6–23% [12–14], which might partially be explained by the application of differing MWI techniques. Furthermore, a reduction and large variation of the MWF in different MS-lesion types, such as contrast enhancing lesions (CEL) and non-enhancing MS-lesions (non-CEL), in comparison to the white matter (WM) of HCs has been reported by a number of recent studies. The reduction of the MWFs in MS-lesions compared to controls thereby demonstrated a substantial heterogeneity from 26%-61% [12,14–16].

Recently, a more noise robust 32 echo myelin water imaging (MWI) processing technique, based on the gold-standard and histopathologically verified protocol by MacKay et al. [11] was developed by Kumar et al. [17], which, in comparison to technically related approaches [11,13], allows for lower SNR, less averages and shorter acquisition times (8 slices, 12 minutes) on a 3T MRI scanner. This major reduction in acquisition time and increased brain coverage makes this sequence available and feasible in larger patient cohorts and trials. Nevertheless, this sequence has not been tested in a clinical setting and larger patient numbers yet.

The purpose of this study was to test this improved technique in a clinical research environment as an add-on to standard imaging. We evaluated age-dependent changes of the MWF in the WM of healthy controls and the NAWM of MS-patients. To further investigate the sensitivity of the utilized MWI technique, we evaluated differences of the MWF between acute and chronic MS-lesions as well as black holes (BH), all representing different degrees of tissue destruction. We hypothesized, that this new technique detects quantitative changes of the MWF in MS-patients compared to HCs and that these changes are comparable with former studies with different techniques and less slices [13–15,18]. We also aimed to investigate the heterogeneity of MWFs in different MS-lesion types with variable degree of tissue destruction.

## Methods

### Patients`characteristics

Seventeen relapsing remitting MS (RRMS) patients diagnosed based on the 2010 revisions of the McDonald criteria [5] and 14 healthy controls were included in our study. The study was approved by the local research Ethical Committee Hamburg (Ethik-Komission der Ärztekammer Hamburg) following the guidelines of the Declaration of Helsinki and written informed consent was obtained from every subject. Patient and control cohorts were age-matched by mean age. All HC individuals were known to be free of physical and mental diseases. Patient’s characteristics are summarized in Table 1.

**Table 1:** Patient´s characteristics: overview of demographic data and clinical parameters of MS-patients and HCs.

Demographic data	HCs	MS-patients
mean age (in years)	34.9 years (r:21–59, sd:13.5)	40.9 years (r:18–64, sd:13.2)
sex (female/male)	n = 10/n = 4	n = 10/n = 7
mean EDSS* (0–10 points)	n/a	2.4 (r: 0–5.5, sd:1.5)
MS-type**	n/a	n = 17 RRMS

* EDSS: Expanded Disability Status Scale compiled for all diseased subjects

** RRMS: Relapsing-Remitting Multiple Sclerosis

HCs = all healthy controls, MS-patients: all MS-patients

sd = standard deviation, r = range, n/a = not applicable

### *In vivo* MRI data acquisition

MRI was conducted on a 3T MR scanner (Skyra, Siemens Medical Systems, Erlangen, Germany) with a 32 channel head and neck coil. Our standard protocol consisted of axial 2D T2w turbo spin echo (TSE) images acquired with TR = 2800ms, TE = 90ms, 43 slices, Matrix: 192x256, slice thickness = 3mm and in plane resolution = 0.5x0.5mm^2^, turbo factor = 5. 3D-FLAIR images with TR = 4700ms, TI = 1800ms, TE = 390ms, 192 slices, slice thickness = 1mm, matrix = 256x256. T1-MPRAGE pre and post Gadolinium with TR = 1900ms, TE = 2.43ms, TI = 900ms, slice thickness = 1mm, matrix = 256x256x192, voxel size = 1x1x1mm and Gd-dose of 0.2ml per kilogram of body weight. In addition to our standard clinical protocol, T2 relaxometry data were acquired using a MESE sequence with the following parameters: echo spacing = 8.3 ms, number of echoes = 32 (maximum TE = 265 ms; TR = 3000 ms), 8 slices, slice thickness = 4 mm, in plane resolution2x2mm^2^, acquisition matrix 128 x 96 with 6/8 partial Fourier, NEX = 4, GRAPPA reduction factor = 2 with 24 reference lines, acquisition time = 12 minutes, slice thickness of refocusing pulse = 12 mm, gap between slices = 4 mm. The 3 times larger refocusing slice thickness was chosen to eliminate non-180° spin refocusing because of the imperfect slice profiles. Only magnitude data were collected.

### Data processing and calculation of MWF maps

Assuming to be in a slow exchange regime where the effect of exchange among various tissue pools (myelin, intra-/extra-cellular water, edema, CSF) can be neglected, the underlying T2-decay can be described by the sum of multi-exponentials. We implemented the multi voxel spatial regularization (MVSR) approach proposed by Kumar et al. [17], where the improved noise robustness of reconstruction is achieved by encoding of the expectation, that volume fractions and T2 relaxation times of tissue compartments change smoothly within coherent brain regions. We preselected fifty different T2 times, which were chosen over a range of 5–600ms on a logarithmic scale. All other data processing steps were identical to the method described by Kumar et al. [17].

### Region of interest and lesion definition

**Fig 1:**
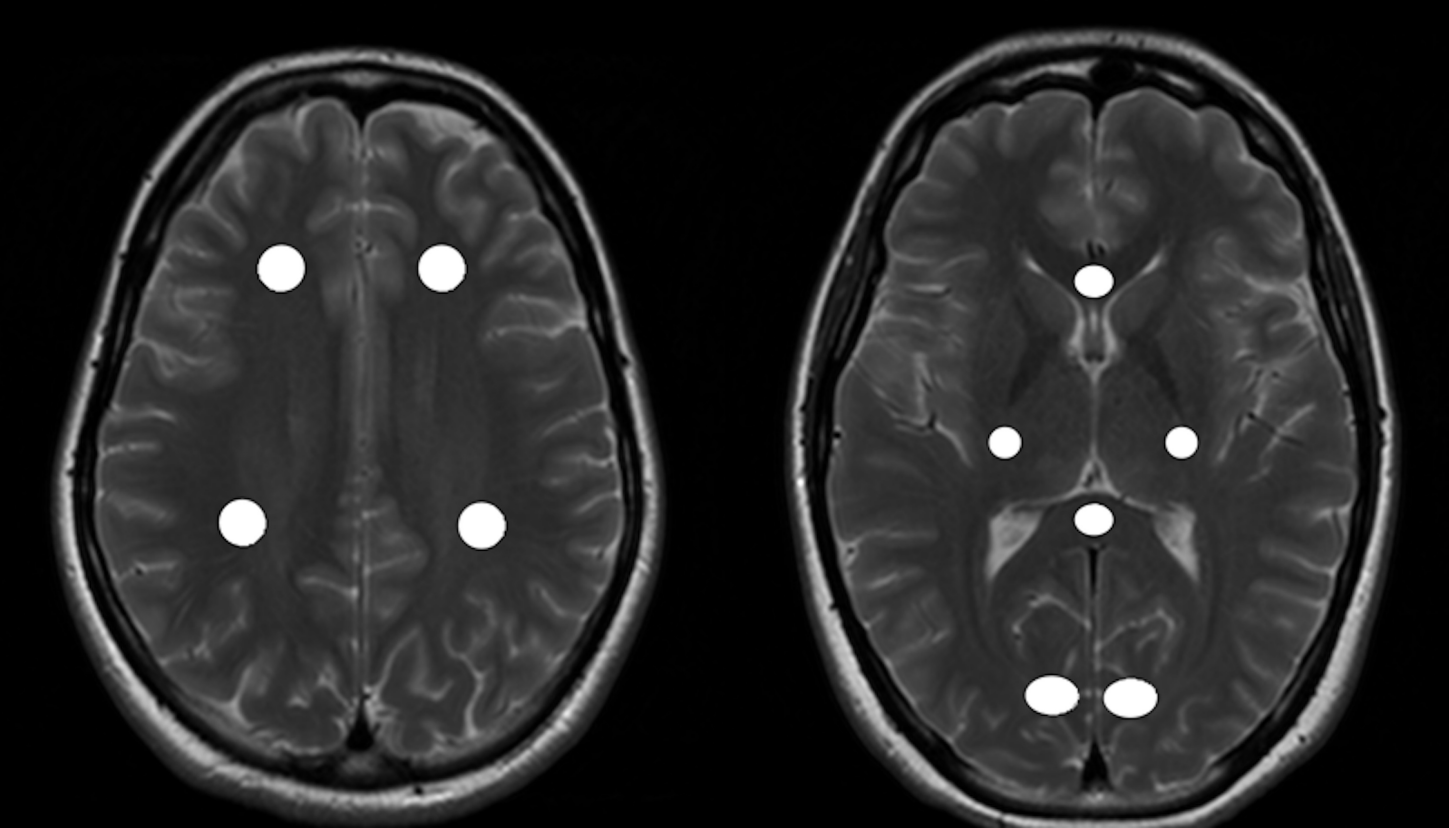
ROI localization in the NAWM. Figure showing regions of interest (ROI) placement on an exemplary T2w image of a healthy control (HC). ROIs have been defined in the normal appearing white matter (NAWM) of both hemispheres in the frontal and parietal NAWM (left side) as well as in the occipital NAWM, the genu and splenium of corpus callosum and cortico-spinal-tractus (right side).

**Fig 2:**
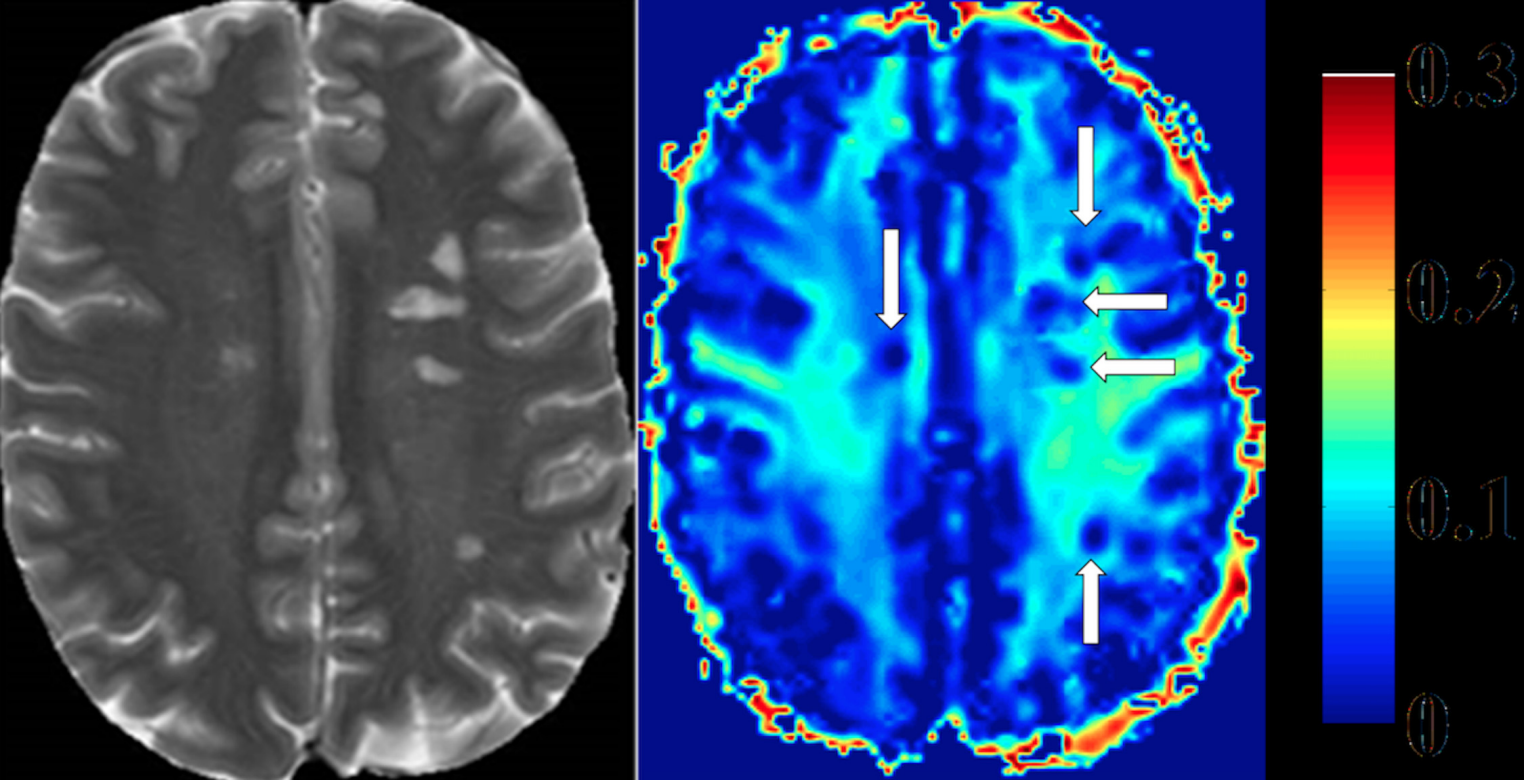
Heat map of myelin water fraction. Left side: T2w image of a Multiple-Sclerosis (MS) patient. Right side: heat map of a myelin water imaging (MWI). T2-hyperintense MS-lesions show clear reductions of myelin water fraction (MWF) (white arrows, right side).

For each individual, ten regions of interest (ROI) were defined in the frontal, parietal and occipital normal appearing white matter (NAWM)/white matter (WM), in the genu (GCC) and splenium (SCC) of corpus callosum and also in the corticospinal tract (CST) (Fig 1) of the corresponding T2w scan, which was then linearly registered to the last echo of the acquired T2 relaxometry data. In addition, MS-lesions were identified in the MS-patient´s group on T2w images and transferred to the last echo of the corresponding acquired T2 relaxometry data. All ROIs were checked for accuracy and manually corrected. All ROIs included a volume of at least four voxels in the myelin water fraction maps (Fig 2). Defined lesions were subdivided into three categories: contrast-enhancing lesions (CEL), black holes (BH) or T2 lesions. Black holes were defined as non-CELs, which appeared hypointense to the cerebral cortex on native T1w images [19]. T2 lesions were regarded as T2 hyperintense lesions that were non-CELs and non-BH lesions. All ROIs were manually defined and outlined using the software ANALYZE 11.0.

### Statistical analysis

Data are presented as mean ± standard deviation (sd) and range. Statistical differences between groups were analyzed with Welch´s t-test, due to different sample sizes and partly non equal estimates of variance. Statistical significance for the results of hypothesis tests was corrected for multiple testing and assumed with an α error of p ≤ 0.05. For age-dependent statistics, (age vs. NAWM/WM MWF) regression analysis and general linear model analysis (GLM) were computed. Correlations between the specific lesion types, sex, Expanded Disability Status Scale (EDSS) and disease-duration (DD) versus the NAWM MWF were computed with GLM and Pearson´s correlation coefficients. Jonckheere-Terpstra-Test was used for variance analysis of different MS-lesions. For data analysis and spreadsheets software packages SPSS (SPSS 21.0) and statistics in R version 3.2.2 were used.

## Results

**Fig 3:**
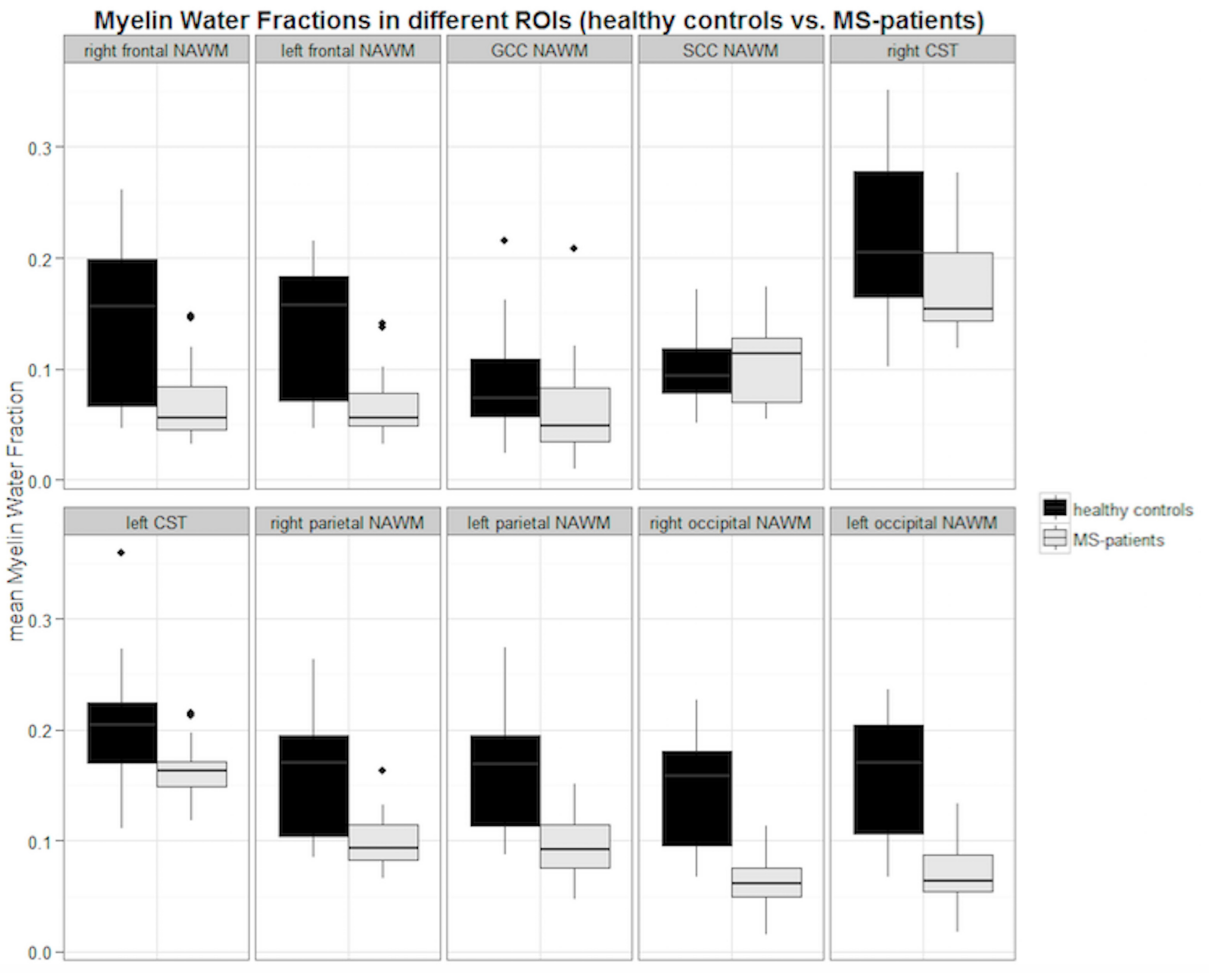
Boxplots of mean MWFs in the NAWM of all MS-patients compared to WM of healthy controls. Black boxes indicate the mean myelin water fraction (MWF) of healthy controls (HC) and grey boxes display the mean MWF of MS-patients in the specific Region of Interest (ROI) localization. NAWM = normal appearing white matter; WM = white matter of healthy controls; GCC = genu of corpus callosum; SCC = splenium of corpus callosum; CST = corticospinal tract.

Comparing the mean MWFs of MS-patients and HCs, we found a significantly decreased MWF in the majority of NAWM ROI locations in MS-patients. Only the mean MWFs measured in the GCC and SCC ROIs were not significantly reduced in MS-patients. Details on the mean MWFs measured in predefined ROIs are listed in Table 2 and Fig 3 visualizes the ascertained results in box plots. In the HC group, we found a mean MWF in WM of 0.15 ± 0.058 over all defined ROIs. In the entire MS-patients group, we measured a mean MWF in the NAWM of 0.10 ± 0.037. The overall mean reduction of the MWF in the NAWM of MS-patients compared to HCs was 37%.

**Table 2:** Mean MWF values in the NAWM of MS-patients and WM of HCs in total study cohort.

ROI localization	MWF of HC	MWF of MS-patients	p-value	mean relative difference
right frontal *	0.142 ± 0.075	0.069 ± 0.039	0.001	51.5%
left frontal *	0.134 ± 0.062	0.069 ± 0.034	0.001	48.5%
GCC	0.089 ± 0.051	0.063 ± 0.041	0.100	29.2%
SCC	0.104 ± 0.034	0.103 ± 0.035	0.656	9.6%
right CST *	0.215 ± 0.073	0.163 ± 0.037	0.037	24.2%
left CST *	0.207 ± 0.064	0.153 ± 0.023	0.017	26.1%
right parietal *	0.160 ± 0.057	0.101 ± 0.027	<0.001	36.9%
left parietal *	0.163 ± 0.056	0.097 ± 0.030	<0.001	40.5%
right occipital *	0.141 ± 0.053	0.063 ± 0.005	<0.001	55.3%
left occipital *	0.153 ± 0.060	0.073 ± 0.033	<0.001	52.2%

Estimated MWFs are listed as mean values ± sd. Regions with significant (p ≤ 0.05) differences of the MWF between HCs and MS-patients group are marked by an asterisk (*).

Legend: NAWM = normal appearing white matter; WM = white matter of healthy controls; HC = healthy controls; CST = corticospinal tract; SCC = splenium of corpus callosum; GCC = genu of corpus callosum; sd = standard deviation.

We found a significant reduction of the mean MWF in all three predefined lesion types compared to the mean MWF in the WM of HCs.

The mean difference of the MWF in CELs, T2-lesions and BHs compared to the MWF in the WM of HCs was 70.2%, 78.0%, and 89.3% respectively.

**Fig 4:**
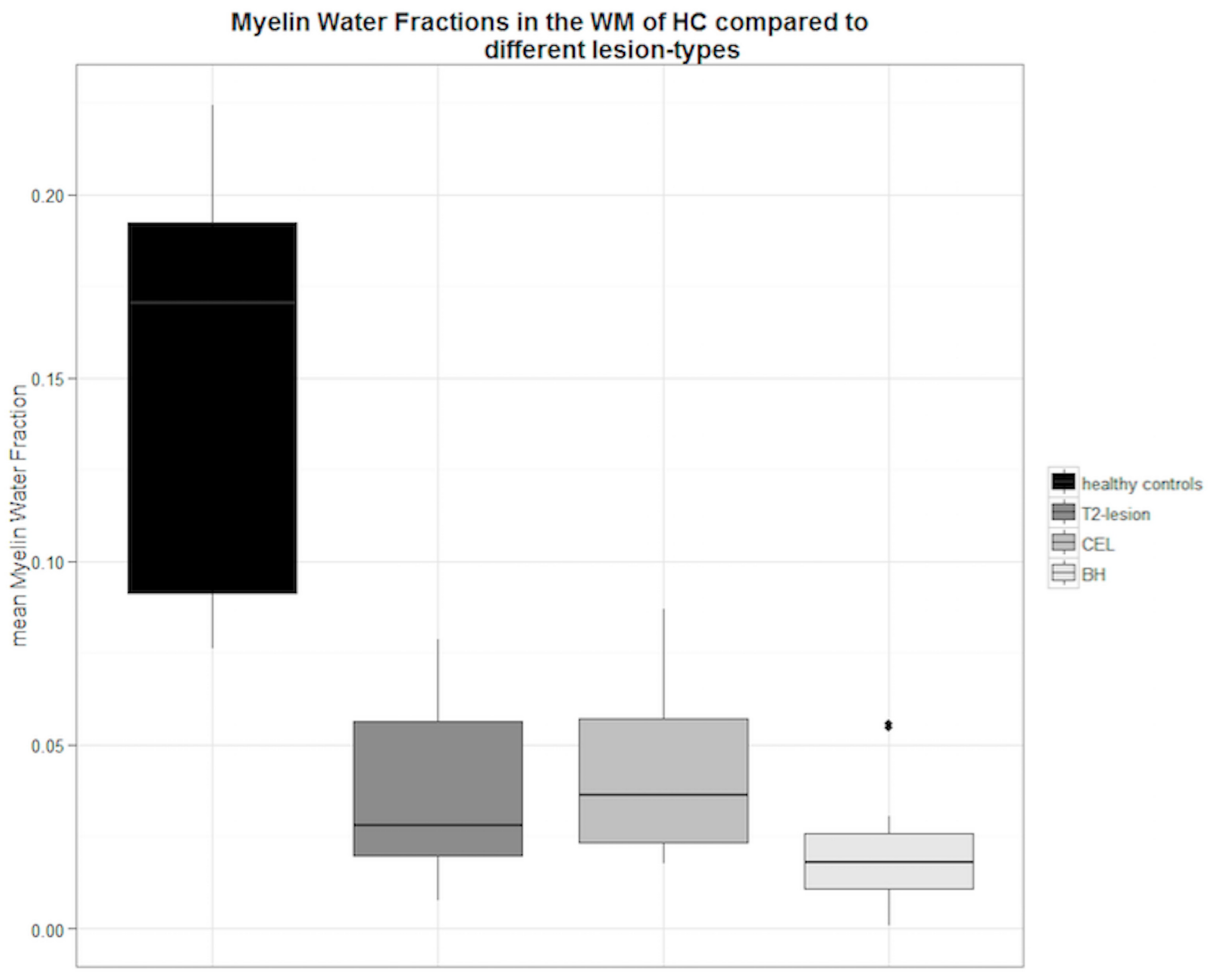
Comparison between different MS-lesion types and the MWF in the WM of all HCs in the study. Boxplot depiction of the mean myelin water fractions (MWF) of different lesion types related to the mean MWF in the white matter (WM) of all healthy controls (HC).

MWF differed significantly between different lesion types (p = 0.02) and showed decreasing values from CEL over T2-lesions to BH. Also a significant correlation was found between all MS-lesion types and the NAWM of MS-patients (R = 0.79, p<0.001). Detailed MWF measurements of the different lesion types are presented in Table 3 and boxplots in Fig 4 display the relationship between MS-lesions and the WM of HCs.

**Table 3:** Mean MWFs in specific MS-lesion types compared to the MWF in the WM of all HCs.

lesion-type	MWF	p-value	mean percentage difference
CEL *	0.044 ± 0.031	<0.001	70.2%
T2-lesion*	0.033 ± 0.018	<0.001	78.0%
BH *	0.016 ± 0.015	<0.001	89.3%

Table 3 shows the mean myelin water fractions (MWF) measurements in the three different lesion types in comparison to the mean MWF in the white matter (WM) of healthy controls (0.15 ± 0.058). Estimated MWFs are listed as mean values ± sd. Significant (p<0.05) differences are marked with asterisks (*). Mean percentage differences between the specific lesion type and the mean white matter MWF of HCs can be seen in the last right column.

Legend: sd = standard deviation; HC = healthy control; CEL = contrast enhancing lesion; BH = black hole.

No age-dependency was found considering the mean MWF in the NAWM of MS-patients (R = 0.16; p = 0.265) and HCs WM (R = 0.09; p = 0.400). DD (r = 0.12; p = 0.305) and EDSS (r = 0.37; p = 0.061) showed no significant correlation with mean MWF in the NAWM as well. GLM also found no significant dependency of age, sex, DD and EDSS with the mean MWF in the NAWM.

## Discussion

We found a significant reduction of the mean MWF in three predefined lesion types of MS-patients, such as CELs, T2 lesions and BHs related to the mean MWF in the WM of HCs. Lesions showed considerable heterogeneity, similar to previous studies [13–15]. We detected a significant reduction of the mean MWF in the NAWM of MS-patients by 24–55% dependent on the location. So far, no other studies evaluated different MS-lesion types and multiple regions of NAWM in patients and healthy controls using this novel multi-slice Bayesian MWI method [17].

Former MESE investigations only utilized a single-slice imaging technique, limiting the spatial detectability of different MS-lesions and inhibiting the assessment of multiple locations of NAWM in different brain regions. Also, a comparable study [13] included a very heterogeneous patient cohort [24 RRMS, 8 Secondary Progressive MS (SPMS) and 1 Primary Progressive MS (PPMS)]. In related T2-prep studies [15], the reduction of the MWF in MS-lesions was smaller, which could also be related to the use of different lesion-masking processing techniques and due to the heterogeneous composition of the included MS-patients (89 patients, 69 with RRMS, 7 SPMS and 13 with clinically isolated syndrome). A recent mcDESPOT investigation [14] provided full-brain MWI scans in a study cohort of 17 PPMS-patients and 17 HCs. However, the authors did not differentiate between any lesion types and the applied technique is, in contrast to multi-echo T2-relaxation models, known to encounter problems with the discrimination of multiple water pools (e.g. long-T2 components), which appeared to be verifiable in MS-lesions and in the NAWM [20,21]. The presence of a long-*T*_2_ component in MS-lesions seems to indicate a different underlying pathology than lesions not exhibiting this phenomenon [22]. Moreover, higher MWF measures are observed using the mcDESPOT method compared to multi-echo T2-relaxometry approaches [14].

However, a reduction and large variation of the MWF in certain MS-lesions in comparison to NAWM has been reported by a number of recent studies. Oh et al. reported a reduced mean MWF in CELs and non-enhancing T2 lesions of 26% and 29% respectively, in comparison to controls. MESE studies by Laule et al. found an average decrease of the MWF in non-specific MS-lesions of 52% compared to the MWF in a similar area of NAWM contralateral to each lesion [13]. Kolind et al. also reported a significant reduction of the MWF in not otherwise specified MS-lesions in comparison to HCs of 61%. Our findings were in line with those of prior studies, once more underlining the capability of MWI in the detection and characterization of MS-lesions. However, the divergent results of all recent observations including ours indicate a wide range of the MWF in MS-lesions, presumably related to the applied MWI-technique, the choice of study-protocol, patient selection, heterogeneous ROI-localizations and lesion-characterizations.

Differences of the MWF values in MS-lesions seem to reflect histopathological heterogeneity and also structural differences in lesion components among the specific MS-lesion types. CELs consist of various cells, such as activated T-cells, macrophages, and microglial cells, which are part of the demyelination process in MS [23,24]. By comparing CELs to non-CE T2-lesions and BHs, we observed the MWF reduction to be at least pronounced in CELs and MWF values were showing a considerable range (2–6%). These findings are similar to former studies. Oh et al. [15,16] only differentiated between CELs and non-CELs. They reported a significant reduction of the MWF in CELs with a wide variation of MWF values (4–13%). This finding was attributed to the concomitant phenomenon of active demyelination and early remyelination processes within the inflammatory lesions. Similar to our study, they also found a more pronounced reduction of the MWF inside non-CE T2-lesions compared to CELs. It has been assumed, that the low specificity of T2-lesions is most likely explained by the several pathological processes occurring at the same time (absence of myelin, partial demyelination, and significant re-myelination) [15], which also might explain the large variance of the MWF measurements inside this lesion type. These findings closely correlate with those of recent publications, which also described a considerable heterogeneity inside of T2 lesions [25]. In addition, lesion characteristics are differing in-between different MS-subtypes. In PPMS-patients, lesions seem to differ in number and underlying pathology [14]. Compared to RRMS-patients, PPMS-patients seem to have fewer brain T2-lesions and less CELs [26]. The lesions are believed to contain fewer inflammatory cells [27], show less acute axonal damage [28] and partly hold extensive remyelination in a large proportion of the lesion area [29], which might rather explain higher MWF values in lesions of PPMS-patients. This is supported by recent DTI and MTR examinations which reported, that MS-patients suffering from PPMS seem to have smaller abnormal changes in the NAWM than patients with RRMS and SPMS [30–33]. However, we found the mean MWF inside of BHs to be the lowest of all evaluated MS-lesion types. This also correlates with histopathological findings demonstrating a severe axonal loss, white matter matrix destruction and widening of the extracellular space in chronic T1-black holes, indicating significant tissue damage [34–36]. Additionally, T1-hypointense lesions show higher water contents, presumably due to a concomitant loss of myelin integrity [37]. This is also supported by MTR-studies, which stated a significant lower MTR in T1-hypointense lesions, as an indicator for possible focal demyelination [37–39]. However, former studies reported no significant differences regarding the myelin water content between isointense T1-lesions and hypointense T1-lesions. Compared to HCs, we also found a modest but noticeable difference of the MWFs in non-black holes compared to black holes of 10%, which might be explained by a potentially higher sensitivity of MWI in the assessment of myelin integrity in MS-lesions.

Additionally, our results revealed a significant reduction of the MWF in NAWM of MS-patients in comparison to HC for most NAWM ROIs (except SCC and GCC). These findings demonstrate that our MWI technique is sensitive not only to large changes in MS due to lesions, but more subtle features of the pathology. Our findings correlated with results from other studies. However, all of these studies used different MWI-techniques and scan protocols, while patient characteristics in these cohorts were inhomogeneous and therefore restrictively comparable. Laule et al. [13] described a 16% reduction of the MWF in the NAWM of a very heterogeneous study cohort. Oh et al. [15] observed a significant decrease of the MWF in the NAWM of MS-patients related to HCs from 6–9%, depending on the individual DD. Kolind et al [14] reported a 6% reduction of the MWF in the NAWM of PPMS-patients related to HCs using mcDESPOT. It is well recognized, that MWF values derived from mcDESPOT are greater than in multi-exponential T2-models [40]. This might be an explanation why we found a considerably higher percentage difference of the MWF in the NAWM of MS-patients related to HCs compared to mcDESPOT. Accordingly, this finding might indicate a higher sensitivity of the here applied MESE-technique to specific demyelinating changes in the NAWM.

In our study, we did not find a significant age-dependency of the MWF in both, healthy and diseased individuals. In contrast, another study reported age-related changes of the white matter integrity in healthy individuals using DTI or MTR imaging [41]. However, the same study also utilized a 3D-GRASE MWI technique, which only showed minor differences of the MWF dependent on age, suggesting this method to be less suitable for the detection of white matter changes especially in young participants. Therefore, the low mean-age of our study cohort might account for the missing link between age and changes of the MWF. Another explanation could be the small number of included patients and the herewith associated statistical imprecision.

Furthermore, we did not find a significant correlation between EDSS or DD with NAWM MWF. The missing link between EDSS, lesion burden and loss of white matter integrity (especially in RRMS-patients) was described in previous studies [15,42]. In contrast, other studies reported a significant correlation between EDSS and a reduction of NAWM MWF [14]. This might underline the importance of the specific MS-subtype for the extend of tissue destruction and clinical impairment reflected by the MWF, while severe progression forms of MS seem to correlate more with EDSS and DD than less progressive subtypes.

Nevertheless, our study is facing some limitations. The proper depiction and the assessment of infratentorial brain regions are still challenging and inhibited by problems with pulsation artefacts and spatial resolvableness, whereas other technical approaches like mcDESPOT are able to provide full brain coverage at the cost of higher acquisition times. While we were able to show the feasibility of our technical approach in a clinical research environment, further investigations with larger study cohorts and longitudinal examinations will be needed to prove the benefit of this MESE-technique in the assessment of disease progression and in the appraisal of therapy effects and outcomes.

## Conclusion

MWF maps based on the fast MESE-technique allow for quantitative assessment of lesions and NAWM in MS. Measurements of the MWF also differentiate between patients and healthy controls, but seem to be independent of age. Different types of MS-lesions showed significant differences in MWF, presumably related to differences in the degree of tissue destruction. Based on this multislice technique, MWF maps appear to be a promising tool for monitoring tissue destruction in clinical MS studies.

## Supporting Information

S1 Dataset MWF values of HCs, MS-patients and MS-lesions. (SAV)
